# Supplementary material for: MetaRibo-Seq measures translation in microbiomes
Source: Nat Commun. 2020 Jun 29;11:3268. doi: 10.1038/s41467-020-17081-z (PMC7324362; doi:10.1038/s41467-020-17081-z)
Supplement: Supplementary file 10 — Supplementary Data 7 [file 41467_2020_17081_MOESM10_ESM.zip › File2/Confidence_High_Taxonomy/73677_out.krona.html]

Javascript must be enabled to view this page.

members
magnitude
magnitudeUnassigned
count
unassigned
taxon
rank

73677\_out

3

superkingdom
2
3

3
976
phylum

200643
3
class

order
3
171549

family
171550
3

3
239759
genus

species
1288121

SRS1041143\_contig\_number\_722SRS105153\_contig\_number\_98SRS147425\_contig\_number\_12138
3
